# Expression of PAFR as Part of a Prosurvival Response to Chemotherapy: A Novel Target for Combination Therapy in Melanoma

**DOI:** 10.1155/2012/175408

**Published:** 2012-04-18

**Authors:** Ana Claudia Onuchic, Camila M. L. Machado, Renata F. Saito, Francisco J. Rios, Sônia Jancar, Roger Chammas

**Affiliations:** ^1^Departamento de Radiologia e Oncologia, Instituto do Câncer do Estado de São Paulo, Faculdade de Medicina, Universidade de São Paulo, 01246-903 São Paulo, SP, Brazil; ^2^Departamento de Imunologia, Instituto de Ciências Biomédicas, Universidade de São Paulo, 05508-900 São Paulo, SP, Brazil

## Abstract

Melanoma cells express the platelet-activating factor receptor (PAFR) and, thus, respond to PAF, a bioactive lipid produced by both tumour cells and those in the tumour microenvironment such as macrophages. Here, we show that treatment of a human melanoma SKmel37 cell line with cisplatin led to increased expression of PAFR and its accumulation. In the presence of exogenous PAF, melanoma cells were significantly more resistant to cisplatin-induced cell death. Inhibition of PAFR-dependent signalling pathways by a PAFR antagonist (WEB2086) showed chemosensitisation of melanoma cells *in vitro*. Nude mice were inoculated with SKmel37 cells and treated with cisplatin and WEB2086. Animals treated with both agents showed significantly decreased tumour growth compared to the control group and groups treated with only one agent. PAFR accumulation and signalling are part of a prosurvival program of melanoma cells, therefore constituting a promising target for combination therapy for melanomas.

## 1. Introduction

Platelet-activating factor (PAF, 1-O-alkyl-2-acetyl-sn-glycero-3-phosphorylcholine) is associated with diverse physiological functions [1–3]. It is produced from membrane glycerophospholipids by enzymatic hydrolysis catalyzed by phospholipase A_2_, which concomitantly generates arachidonic acid that is later converted to precursors of eicosanoids [1, 4]. PAF is secreted by many different cell types, including endothelial, stromal, and inflammatory cells, as well as platelets and keratinocytes. It has also been described to be produced by many different tumour cells [5–7]. PAF is not stored in cells and its synthesis relies mainly on cell activation, occurring in response to different stimuli, such as growth factors, PAF agonists, ultraviolet light irradiation, and oxidative stress, including stress induced by chemotherapy [1, 6].

PAF acts via its membrane-associated receptor in responsive cells [4]. PAF receptor (PAFR) is a seven transmembrane-spanning G-protein coupled receptor expressed in several cell types, such as endothelial and inflammatory cells, and also in tumour cells. Engagement of PAFR induces phosphatidylinositol turnover, a rise in intracellular calcium, and activation of kinases and antiapoptotic pathways [8–10]. The binding of PAF to its receptor results in the transcription of several genes encoding cytokines such as IL-6, -8, -10, TNF-*α*, and VEGF, as well as COX-2 and iNOS. It also elicits eicosanoid and PAF production. Interestingly, different angiogenic factors, such as VEGF and FGF, induce PAF synthesis in a positive feedback loop [1, 6, 11, 12].

The role of PAF/PAFR in tumours has been investigated in recent years. PAF has been associated with early malignant transformation in BRCA1-mutant epithelial ovarian cells [13], while melanocytic tumorigenesis has been observed in transgenic mice overexpressing PAFR [14]. In tumours, PAF has also been described to negatively modulate the local immune system, inhibiting Th1 responses and promoting tumour growth [1, 11]. Intratumoural production of PAF has been attributed to both tumour cells and microenvironmental cells, such as infiltrating macrophages, and has been shown to be responsible for potentiating tumour growth, neoangiogenesis, and cell motility [12, 15]. The many effects of PAF in tumours, such as increased vascular permeability, induction of neoangiogenesis, and activation of metalloproteinases, along with its systemic effects, including increased cell adhesion to endothelia, have reinforced the concept that PAF promotes tumour metastasis [1, 5].

Recent experiments have shown an important role of PAF in chemotherapy, demonstrating that PAFR activation can augment cytokine production induced by chemotherapy through a mechanism dependent on NF-*κ*B [16]. Despite contrary reports [6], recent data have shown that PAFR activation induces upregulation of antiapoptotic gene products, such as Bcl-2, thus attenuating the cytotoxic effect of chemotherapeutic agents [9]. In this scenario, specific PAFR antagonists may have a potential effect in blocking protective tumour responses and potentiating chemotherapy [17–19].

Earlier experiments from our group have shown that the PAFR antagonist WEB2170 inhibits tumour growth in a murine melanoma model, improving overall survival when combined with chemotherapy [20]. This led us to investigate the role of PAFR in human melanoma cell lines and evaluate the mechanisms of microenvironmental response and possible inhibition of tumour growth in a combination therapy with a similar PAFR antagonist, WEB2086.

## 2. Materials and Methods

### 2.1. SKmel37 Human Melanoma Cells

The human melanoma line SKmel37 was cultured in minimal essential medium Eagle (MEM) (Gibco, Invitrogen, Carlsbad, CA, USA), pH 7.2 supplemented with 10% fetal bovine serum (Gibco, Invitrogen, Carlsbad, CA, USA), in the absence of antibiotics, at 37°C and 5% CO_2_.

### 2.2. *In Vitro* Experiments with SKmel37

SKmel37 cells were cultured *in vitro* and treated with cisplatin (concentrations of 2.5 and 5.0 *μ*M) and/or WEB2086 (5 and 20 *μ*g/mL) and/or PAF (3 *μ*g/mL, *β*-acetyl-*γ*-O-hexadecyl-L-*α*-phosphatidylcholine, Sigma-Aldrich, St. Louis, MO, USA). After treatment, the cells were washed twice with phosphate-buffered saline (PBS) at room temperature (RT) and removed with a cell scraper in a protein lysis buffer (1% Triton X-100, 1% sodium deoxycholate deocolate, 150 mM NaCl, 1% sodium dodecyl sulphate (SDS), 10 mM NaF, 50 mM Tris-HCl_2_ *μ*g/mL aprotinin, 1 mM PMSF (phenylmethanesulphonyl fluoride) and 1 mM sodium orthovanadate for protein extraction. Cells were then collected in a PBS-EDTA and 0.25% trypsin solution (Gibco, Invitrogen, Carlsbad, CA, USA) for flow cytometric experiments.

### 2.3. Animals

Twelve-week-old female nude mice received a subcutaneous inoculation of 1 × 10^6^ SKmel37 human melanoma cells in the right flank. Animals were submitted to all procedures in a laminar hood and housed in groups of 7 individuals in ventilated cages (Alesco, Campinas, SP, Brazil). Animals were maintained in ventilated racks with sterilised food and water *ad libitum*, in a 12/12 h light/dark cycle. All procedures were in accordance with the ethical principles adopted by the Brazilian College of Animal Experimentation and approved by the Ethical Committee for Animal Research of the Faculdade de Medicina da Universidade de São Paulo (procedure approval number 0993/09).

### 2.4. Evaluation of Tumour Growth

SKmel37 cells (1 × 10^6^ cells) were injected subcutaneously in nude mice and tumour growth was determined by measuring the diameter of the solid tumour mass, from which the volume was estimated using the formula for a spheroid: *V* = 0.52 × (largest axis) × (smallest axis)^2^.

### 2.5. Treatments

Nude mice were divided into 4 groups of 7 animals and received the following treatments: no treatment (control group), only cisplatin, only WEB2086, and cotreatment of cisplatin with WEB2086. The PAFR antagonist WEB2086 (5 mg/kg; Tocris, Ellisville, Missouri, USA) was injected intraperitoneally (i.p.) seven days after tumour implantation (when more than half of the animals had palpable tumours) and repeated 14 and 21 days after the implantation. Animals were treated concomitantly with cisplatin (2 mg/kg, Sigma-Aldrich, St. Louis, MO, USA). The control groups received the same volume of the dilution vehicle (PBS for cisplatin; PBS + DMSO at 0.3% for WEB2086). Animals were sacrificed one day after the third cycle of treatment (22 days after inoculation) in a carbon dioxide (CO_2_) chamber in an increased CO_2_ degree machine program until all animals were euthanised.

### 2.6. Human PAFR Gene Expression

RNA was isolated using TRIzol reagents (Life-Technologies). For the real-time reverse-transcriptase polymerase chain reaction (PCR), cDNA was synthesized using the RevertAid First Strand cDNA Synthesis Kit (Fermentas Life Sciences, Ontario, USA), according to the manufacturer's instructions. PCR-master mix (Power SyBr Green, Applied Biosystems, Warrington, UK) containing the specific primers were then added. hPAFR sense primer GGGGACCCCCATCTGCCTCA and anti-sense primer GCGGGCAAAGACCCACAGCA and GAPDH sense primer GAGTCAACGGATTTGGTCGT and anti-sense primer TTGATTTTGGAGGGATCTCG. Real-time PCR was performed using a Stratagene Mx3005P QPCR Systems (Santa Clara, CA, USA). Relative gene expression of hPAFR and GAPDH were calculated by 2^(−ΔΔC(T))^. Data are shown in fold increase related to untreated cells.

### 2.7. Western Blotting

Total protein extraction was carried out after cells were homogenised in 1% Triton X-100, 1% sodium deoxycholate, 150 mM NaCl, 1% SDS, 10 mM NaF, 50 mM Tris-HCl, 2 *μ*g/mL aprotinin, 1 mM PMSF, and 1 mM sodium orthovanadate. To remove insoluble materials, centrifugation (13,000 g centrifugation, 4°C for 15 minutes) was performed. Protein concentration was determined using the bicinchoninic acid method. Total protein extracts from each sample were eletrophoretically separated in SDS-PAGE and electroblotted onto a PVDF membrane according to standard procedures. Membranes were blocked with PBS-Tween containing 5% nonfat dry milk and then incubated with an anti-PAF receptor polyclonal antibody (rabbit anti-mouse PAFR, Cayman Chemical) and anti-*β*-actin mouse monoclonal antibody (Sigma-Aldrich) in PBS-Tween containing 1% BSA for 12 h at 4°C. Controls for reactivity of anti-PAF receptor antibody included western blottings of protein extracts from macrophages derived from wild-type (positive control) and *PAFR* null mice (negative control). Membranes were washed with PBS-Tween and incubated with a specific horseradish peroxidase- (HRP-) labelled secondary antibody (Sigma-Aldrich, 1 : 5,000). Reactive bands were detected with luminol and H_2_O_2_ using an image capture system (Image Quant LAS 4000, GE Health care).

### 2.8. Flow Cytometry

After treatment, cells were fixed in 70% ethanol for at least 2 h, washed twice in PBS (5 min, 600× g), and then incubated with 200 *μ*L of a propidium iodide (PI) solution containing 20 *μ*g/mL of PI (Sigma, St. Louis, MO, USA), 200 *μ*g/mL RNAse A (Invitrogen Biotechnology, Carlsbad, CA, USA), and 0.1% Triton v/v in PBS. After 30 min of incubation in the dark, data were acquired in a FACSCALIBUR flow cytometer (BD Biosciences, San Jose, CA, USA). Samples were analysed using the Cell Quest Pro software, and the results were expressed in the percentage of hypodiploid cells and those in the G0/G1, S, and G2/M phases.

### 2.9. Statistical Analyses

Data were analysed using one-way ANOVA followed by Bonferroni post hoc tests using GraphPad Prism 4.0 software. Differences were considered significant when *P* < 0.05.

## 3. Results and Discussion

### 3.1. SKmel37 Cells *In Vitro* Express PAFR, and Treatment with Cisplatin Induces Increased PAFR Expression

**Figure 1:**
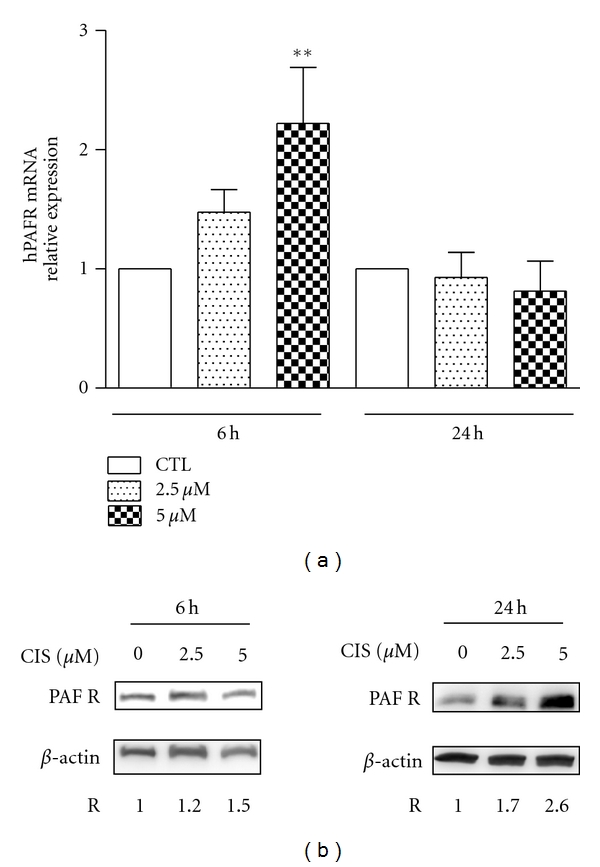
Cisplatin-treated SKmel37 cells accumulate PAFR. (a) qPCR analysis of PAFR expression in SKmel37 cells exposed to cisplatin for 6 and 24 hours, relatively to expression levels of GAPDH. PAFR expression was significantly increased in cells treated with 5 mM cisplatin after 6 hours, but not in any other condition, as compared to the control (CTL, no treatment; **P < 0.01). (b) Western blot analysis of *in vitro* cisplatin-treated SKmel37 cells using anti-PAFR and anti-*β*-actin antibodies. At 6 h after treatment, cells exposed to cisplatin did not reveal significant increase in PAFR protein expression, while at 24 h, cells treated with increasing concentrations of cisplatin presented an up to 2.6-fold increase in PAFR levels.

SKmel37 cells were cultured *in vitro* and treated with cisplatin (2.5 and 5.0 *μ*M) for 6 and 24 h. PAFR expression was evaluated by qPCR and its accumulation analyzed by Western blotting and flow cytometric experiments. Control cells received no treatment. However, when treated with cisplatin, SKmel37 cells had increased expression of PAFR (Figure 1), observed both at the RNA level, upon 6 h of cisplatin treatment, and at the protein level, after 24 h of treatment (Figures 1(a) and 1(b)). Specificity of the polyclonal antibody to PAFR was ascertained by lack of specific labeling in western blottings of protein extracts of macrophages derived from PAFR knock-out mice (data not shown). These results show that PAFR is present in SKmel37 cells, and its expression is induced by exposure to the chemotherapeutic agent cisplatin in a dose-dependent manner (up to 5 *μ*M). Increase in the expression of the PAFR within six hours of treatment suggests *de novo* expression of the PAFR gene and subsequent accumulation of its product along the first 24 hours of treatment. It has been shown that cisplatin induces endoplasmic reticulum stress [21], a switch between an autophagic prosurvival response and an apoptotic response. Apoptosis tends to occur at later time points (such as 24 hours). The cellular response to cisplatin may explain the drop of mRNA levels for the PAFR gene after 24 hours of cisplatin exposure, as observed in Figure 1(a). These data strengthen the proposed role of PAFR as part of a protective response to cell damage and cell death [22], such as that caused by chemotherapy, and indicate that the SKmel37 cell line might be susceptible to the effects of blocking the PAFR pathway by inhibiting the tumour cell response to stress and subsequently enhancing chemotherapy efficacy.

### 3.2. PAF Protects SKmel37 Cells from Cell Death Induced by Cisplatin *In Vitro*, and the PAFR Antagonist WEB2086 Enhances Cisplatin-Induced Cell Death in the Presence of PAF

SKmel37 cells were cultivated *in vitro *and treated with cisplatin (5 *μ*M) and WEB2086 (5 *μ*g/mL) for 24 h, in the presence or absence of PAF (3 *μ*mol/L, based on experimental delineation in Seo et al. [9]). WEB2086 was initially added to the culture medium, and after 30 minutes, cisplatin and/or PAF were added. After 24 hours, cells were harvested with trypsin. Distribution of cells within different phases of the cell cycle and cell death were assessed by propidium iodide staining. Data were analysed with one-way ANOVA and Bonferroni posttests.

**Figure 2:**
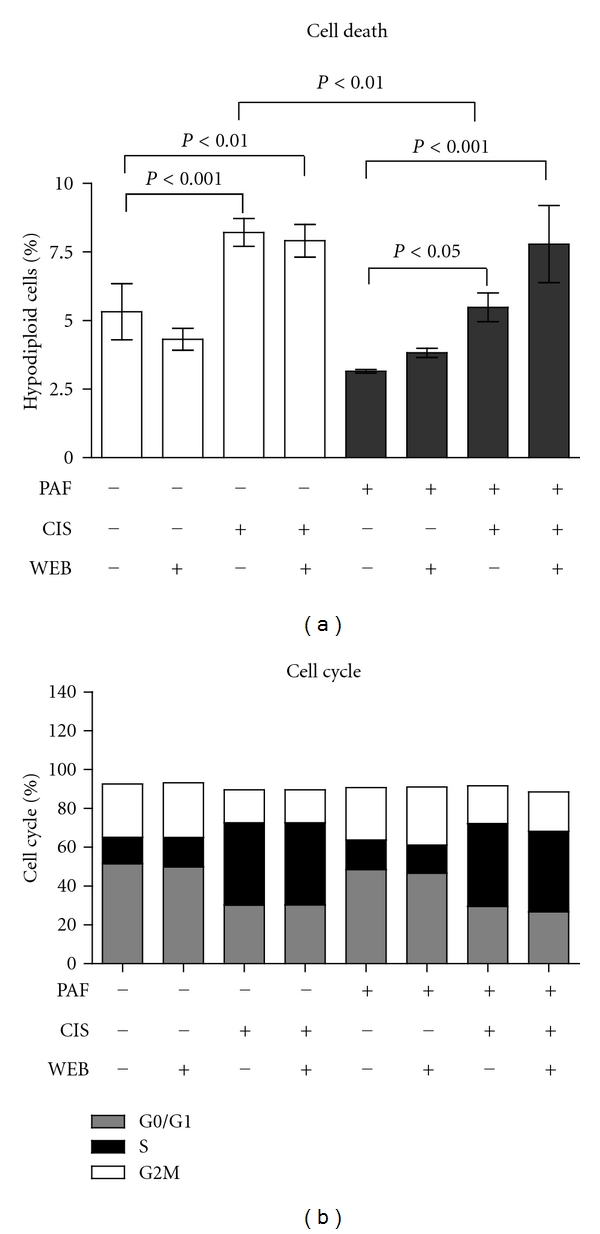
PAF protects SKmel 37 cells from cisplatin-induced cell death *in vitro*. (a) Cell death was measured as the percentage of hypodiploid cells (in total cell population) at each condition. (b) Cell cycle analysis of the live cell population in each condition. CIS: cisplatin; WEB: WEB2086.

In the absence of PAF in the culture medium, WEB2086 had no effect on cell death, while only cisplatin caused a significant increase in cell mortality that was not affected by WEB2086 (Figure 2(a)). When PAF was added to the cell culture, there was a significant decrease in cell death, indicating that PAF has a protective effect on tumour cells. However, there was a clear increase in cell death when cells were exposed to cisplatin and WEB2086, compared to cisplatin alone, although this was not statistically significant. The presence of PAF in the culture medium more closely resembles the *in vivo *condition (in which PAF is present in the microenvironment, produced by tumour and/or microenvironmental cells). These results further support the idea that PAFR-dependent pathways may be involved in a protective response to cell damage and when blocked, cell death induced by chemotherapeutic treatment increases. Indeed, cells treated with cisplatin in the presence of PAF showed a significant decrease in cell death, compared to cells treated with cisplatin in the absence of PAF, thus suggesting that PAF may protect SKmel37 cells from cisplatin-induced death. This led us to investigate the potential effect of PAFR antagonism in an *in vivo* model subjected to chemotherapy.

Cell cycle analysis demonstrated the effectiveness of treatment with cisplatin, showing an accumulation of cells in S/G2/M (Figure 2(b)). Interestingly, exposure to PAF or WEB2086 alone did not cause significant changes in the cell cycle, as observed in normal nonmalignant retinal cells, in which PAF causes an arrest of the cell cycle at the S/G2 transition [23].

### 3.3. Combination Therapy with Cisplatin and WEB2086 in Melanoma-Bearing Mice Slows Tumour Progression and Increases Tumour Regression

**Figure 3:**
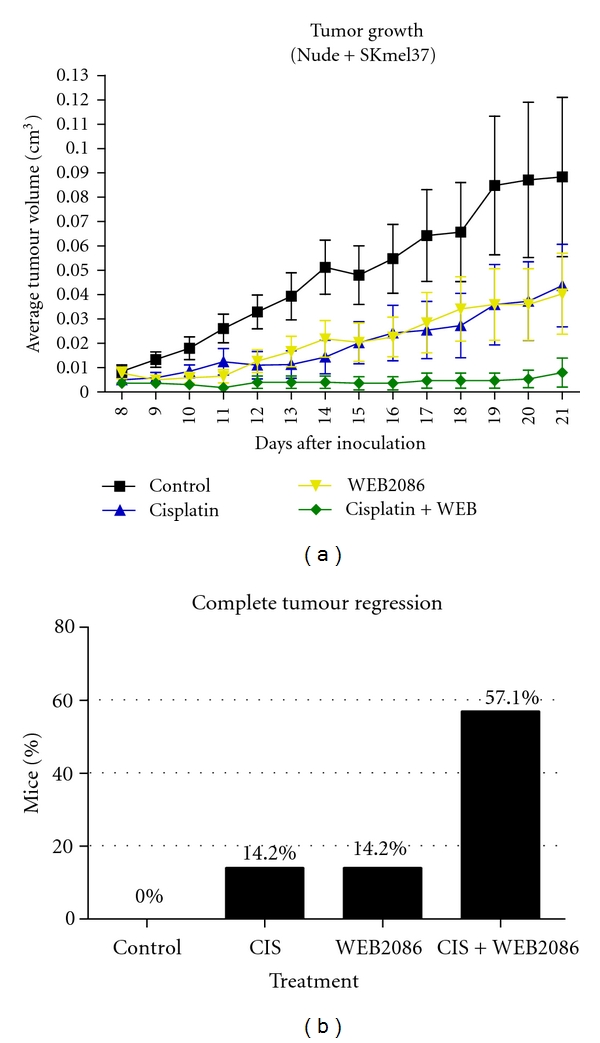
Combination therapy using cisplatin and the antagonist of PAFR is more efficient than either therapy alone (a) Average tumour volume in each experimental group (±SEM) measured daily. (b) Percentage of complete tumour regression in each experimental condition.

SKmel37 cells (1 × 10^6^) were injected subcutaneously into nude mice, and tumours were measured daily with a caliper. Tumour volume was calculated by the formula: maximum diameter × (minimum diameter)^2^  × 0.52. Cisplatin (2 mg/kg) and/or WEB2086 (5 mg/kg) were injected intraperitoneally every 7 days after the tumour cell injection, for a total of 3 cycles of treatment. Animals were sacrificed and had their tumours excised one day after the last cycle of treatment. The growth curves (Figure 3(a)) showed a slight decrease in tumour volume in mice treated with either cisplatin or WEB2086, in comparison to the control group; however, the most important finding was a significant inhibition of tumour growth in the group submitted to the combination therapy (cisplatin and WEB2086). This group showed the highest percentage of complete tumour regression (accounted as tumours that reached a minimum size of 2 mm × 2 mm and afterwards, during the course of therapy, disappeared completely, not being detected macroscopically through inspection/palpation or during animal dissection, as shown in Figure 3(b)). These data suggest that combination therapy was effective not only in slowing tumour growth, but also in reducing and possibly eliminating tumours.

These results show that part of the antitumour effect of WEB2086 may result from a direct effect on tumour cells, potentiated by the increased expression of PAFR via cisplatin, thus making tumour cells more susceptible to PAFR antagonism and also supporting the presence of PAF in the tumour microenvironment. Interestingly, the decrease in cell proliferation observed with cotreatment in this *in vivo* model was significant, different to what was observed in the *in vitro* model. This suggests that tumour growth inhibition and cell death are not only the consequences of direct PAFR antagonism in tumour cells, but also probably results from the effect of PAFR antagonism in other microenvironmental cell types, such as endothelial cells (especially in the context of neoangiogenesis inhibition) and infiltrating inflammatory cells.

## 4. Conclusions and Perspectives

The observation that chemotherapy caused an increase in PAFR expression in human melanoma SKmel37 cells indicated the possible participation of PAFR in tumour response mechanisms, such as stress responses caused by chemotherapeutic agents. Here we provided empirical evidence that the PAFR pathway is involved in a protective response to cell damage and its blockage favors chemotherapy-induced cell death.

 There is now increasing evidence for activation of cytosolic calcium-independent phospholipase A2 (iPLA2) in the microenvironment of tumors subjected to therapy, for example, radiotherapy, in a caspase-3-dependent manner [24]. Activation of iPLA2 led to increased production of arachidonic acid and subsequent PGE2 production. At the same time, iPLA2 activity favors accumulation of the PAF precursor. We had previously shown that presence of apoptotic cells within the tumor microenvironment favored tumor growth [25], a process known as tumor repopulation [24]. Our results suggest that activation of PAFR pathways may support tumor survival and therefore tumor repopulation in melanomas. These results are of potential interest as they widen the field of investigation into the roles of PAFR and also support the concept that PAFR antagonists may be useful for adjuvant therapy, improving the efficacy of chemotherapy.

## References

[1] Melnikova VO, Villares GJ, Bar-Eli M (2008). Emerging roles of PAR-1 and PAFR in melanoma metastasis. *Cancer Microenvironment*.

[2] Demopoulos CA, Pinckard RN, Hanahan DJ (1979). Platelet-activating factor. Evidence for 1-0-alkyl-2-acetyl-sn-glyceryl-3-phosphorylcholine as the active component (a new class of lipid chemical mediators). *The Journal of Biological Chemistry*.

[3] Benveniste J, Tencé M, Varenne P, Bidault J, Boullet C, Polonsky J (1979). Semi-synthesis and proposed structure of platelet-activating factor (P.A.F.): PAF-acether an alkyl ether analog of lysophosphatidylcholine. *Comptes Rendus des Seances de l’Academie des sciences. Serie D*.

[4] Prescott SM, Zimmerman GA, McIntyre TM (1990). Platelet-activating factor. *The Journal of Biological Chemistry*.

[5] Braeuer RR, Zigler M, Villares GJ, Dobroff AS, Bar-Eli M (2011). Transcriptional control of melanoma metastasis: the importance of the tumor microenvironment. *Seminars in Cancer Biology*.

[6] Li T, Southall MD, Yi Q (2003). The epidermal platelet-activating factor receptor augments chemotherapy-induced apoptosis in human carcinoma cell lines. *The Journal of Biological Chemistry*.

[7] Camussi G, Montrucchio G, Lupia E, Soldi R, Comoglio PM, Bussolino F (1997). Angiogenesis induced *in vivo* by hepatocyte growth factor is mediated by platelet-activating factor synthesis from macrophages. *Journal of Immunology*.

[8] Honda ZI, Ishii S, Shimizu T (2002). Platelet-activating factor receptor. *Journal of Biochemistry*.

[9] Seo SK, Ko HM, Kim HA (2006). Platelet-activating factor induces up-regulation of antiapoptotic factors in a melanoma cell line through nuclear factor-*κ*B activation. *Cancer Research*.

[10] Kitagawa D, Taketomi A, Kayashima H (2008). Expression of platelet-activating factor receptor: a novel prognosticator in patients with hepatocellular carcinoma following hepatectomy. *Oncology*.

[11] Melnikova VO, Balasubramanian K, Villares GJ (2009). Crosstalk between protease-activated receptor 1 and platelet-activating factor receptor regulates melanoma cell adhesion molecule (MCAM/MUC18) expression and melanoma metastasis. *The Journal of Biological Chemistry*.

[12] Bussolati B, Biancone L, Cassoni P (2000). PAF produced by human breast cancer cells promotes migration and proliferation of tumor cells and neo-angiogenesis. *American Journal of Pathology*.

[13] Zhang L, Wang D, Jiang W (2010). Activated networking of platelet activating factor receptor and FAK/STAT1 induces malignant potential in BRCA1-mutant at-risk ovarian epithelium. *Reproductive Biology and Endocrinology*.

[14] Ishii S, Nagase T, Tashiro F (1997). Bronchial hyperreactivity, increased endotoxin lethality and melanocytic tumorigenesis in transgenic mice overexpressing platelet-activating factor receptor. *The EMBO Journal*.

[15] Biancone L, Cantaluppi V, del Sorbo L, Russo S, Tjoelker LW, Camussi G (2003). Platelet-activating factor inactivation by local expression of platelet-activating factor acetyl-hydrolase modifies tumor vascularization and growth. *Clinical Cancer Research*.

[16] Darst M, Al-Hassani M, Li T (2004). Augmentation of chemotherapy-induced cytokine production by expression of the platelet-activating factor receptor in a human epithelial carcinoma cell line. *Journal of Immunology*.

[17] Cellai C, Laurenzana A, Vannucchi AM (2006). Growth inhibition and differentiation of human breast cancer cells by the PAFR antagonist WEB-2086. *British Journal of Cancer*.

[18] Cellai C, Laurenzana A, Bianchi E (2009). Mechanistic insight into WEB-2170-induced apoptosis in human acute myelogenous leukemia cells: the crucial role of PTEN. *Experimental Hematology*.

[19] Tsoupras AB, Iatrou C, Frangia C, Demopoulos CA (2009). The implication of platelet activating factor in cancer growth and metastasis: potent beneficial role of PAF-inhibitors and antioxidants. *Infectious Disorders*.

[20] de Oliveira SI, Andrade LNS, Onuchic AC (2010). Platelet-activating factor receptor (PAF-R)-dependent pathways control tumour growth and tumour response to chemotherapy. *BMC Cancer*.

[21] Rovetta F, Stacchiotti A, Consiglio A (2012). ER signaling regulation drives the switch between autophagy and apoptosis in NRK-52E cells exposed to cisplatin. *Experimental Cell Research*.

[22] Bazan HEP, Ottino P (2002). The role of platelet-activating factor in the corneal response to injury. *Progress in Retinal and Eye Research*.

[23] Fragel-Madeira L, Meletti T, Mariante RM (2011). Platelet activating factor blocks interkinetic nuclear migration in retinal progenitors through an arrest of the cell cycle at the S/G2 transition. *PLoS One*.

[24] Huang Q, Li F, Liu X (2011). Caspase 3-mediated stimulation of tumor cell repopulation during cancer radiotherapy. *Nature Medicine*.

[25] Correa M, Machado J, Carneiro CRW (2005). Transient inflammatory response induced by apoptotic cells is an important mediator of melanoma cell engraftment and growth. *International Journal of Cancer*.

